# Supplementary material for: Household water and food insecurity negatively impacts self-reported physical and mental health in the Vietnamese Mekong Delta
Source: PLoS One. 2022 May 5;17(5):e0267344. doi: 10.1371/journal.pone.0267344 (PMC9071150; doi:10.1371/journal.pone.0267344)
Supplement: S1 Checklist — (DOCX) [file pone.0267344.s001.docx]

Inclusivity in global research

PLOS’ policy on inclusivity in global research aims to improve transparency in the reporting of research performed outside of researchers’ own country or community and ensures that PLOS publications reporting global research adhere to high standards for research ethics and authorship. Authors of relevant research articles may be asked to complete the questionnaire below, which outlines ethical, cultural, and scientific considerations specific to inclusivity in global research. This questionnaire may be requested when researchers have travelled to a different country to conduct research, if research uses samples collected in another country, research with Indigenous populations or their lands, or if research is on cultural artefacts. Researchers travelling to another country solely to use laboratory equipment will not normally be required to complete the questionnaire. However, the questionnaire can be requested at the journal’s discretion for any submission – if you have been requested to complete this questionnaire by the PLOS journal you submitted to, please do so.

Please complete the questionnaire below and include this as a Supporting Information file with your manuscript. Note that if your paper is accepted for publication, this checklist will be published with your article in the supporting information files. Please ensure that you reference the checklist in the main body of your manuscript. We suggest adding a subsection ‘Inclusivity in global research’ to your Methods section and adding the following sentence: “Additional information regarding the ethical, cultural, and scientific considerations specific to inclusivity in global research is included in the Supporting Information (S1 Checklist)”

The questions have been designed to be applicable to a wide range of study types, and there are subsections for both human subjects research and non-human subjects research. If any of the questions are not relevant to your research please mark them as “N/A” as appropriate.

**Ethical considerations, permits and authorship**

*This section is applicable to all research types.*

Provide details as to who granted permissions and/or consent for the study to take place in the Methods section of your manuscript. This should include the names of **all** ethics boards, governmental organizations, community leaders or other bodies that provided approval for the study. If individuals provided approval refer to these people by their role or title but do not list their name(s).

Ethics approval for this study was obtained from the Queensland University of Technology Human Research Ethics Committee, Australia (QUT UHREC 1700000907) and the Institute of Public Health of Ho Chi Minh City, Vietnam (122/TB-VYTCC). Additionally, we received consent for this study to take place from Heads of the Provincial Medicine Centers (PMCs) of the two provinces the study was conducted.

Details regarding permissions and consent for the study to take place in the Methods section of our manuscript were reported on page number 3.

If there were any deviations from the study protocol after approval was obtained please provide details of these changes in the Methods section of your manuscript.
Did this study involve local collaborators that are residents of the country where the research was conducted or members of the community studied? If you do not have any authors from said communities, please provide an explanation for this below. Everyone listed as an author should meet PLOS’ criteria for authorship and all individuals who meet these criteria should be included in the author byline, rather than the acknowledgements. Authorship criteria is based on the International Committee of Medical Journal Editors (ICMJE) Uniform Requirements for Manuscripts Submitted to Biomedical Journals - for further information please see here: <https://journals.plos.org/plosone/s/authorship>.

This research was conducted in two rural provinces of the Southern Vietnam. The principal researcher of this study is Vietnamese. She was a PhD candidate of QUT and also is the first author of this paper. In the time of conducting this research, she collaborated with local health staff regarding logistics but not involving in designing the research or writing the manuscript. Thus, we acknowledged the assistance of the local staff in the manuscript but did not list them as authors of the manuscript.

N/A

**Human subjects research (e.g. health research, medical research, cross-cultural psychology)**

Did you obtain written informed consent from a representative of the local community or region before the research took place? How did you establish who speaks for the community? Details of written informed consent obtained from study participants should be reported separately in the Methods section of your manuscript.

Before commencing the study, we obtained written informed consents from the Heads of PMCs of the two provinces where this study was conducted. We sent them all related documents, such as the proposed research, ethical concerns for participants and procedures to manage risks which can be arised when the study was conducted, to ensure that they can understand the objectives and methods of the research. We also hold a one day meeting with representatives of the PMCs to share the prosed research and collected their recommendations to improve the ease of the comprehension and understanding for local participants.

The heads of the PMCs then introduced the research team their staff who were then the representatives to speak to selected households regarding objectives of the research and invited households to take part in the study. Details of written informed consent obtained from study participants were reported separately in the Methods section on page 3 of our manuscript.

How did members of the local community provide input on the aims of the research investigation, its methodology, and its anticipated outcome(s)? When engaging with the local community, how did you ensure that the informed consent documents and other materials could be understood by local stakeholders?

Before commencing the research, the research team conducted a one day meeting with all local stakeholders. The main purpose of the meeting was to present the stakeholders the objectives and methodologies of the research as well as ethical concersn such as risks which can be arised when the study was conducted. The research team also provided strategies to manage these potential risks. In this meeting, the local stakeholders also provided their opinions regarding ease of comprehension and recommendations for improving the words and programs to be suilatble with local cultural context for easier understanding of the informed consent documents and other materials.

Additionally, one day before the research team visited the selected households, the researcher requested local staff to approach households in advance to see whether they are willing to allow local staff to provide their names to the researcher. The local staff also provided households detailed information about the research so that they can contact with the researcher directly if they have any concerns regarding the research. During the household interviews, there was always a local staff who accompanied with research team to assist them if any language barriers occurred.

Will the findings of the research be made available in an understandable format to stakeholders in the community where the study was conducted (e.g. via a presentation, summary report, copies of publications, etc.)? Please provide details of how this will be achieved.

We delivered summary reports of the main findings to the stakeholders via email. If the stakeholders had any questions regarding the findings, we provided them answers.

The primary objectives of the research was to investigate household food insecurity and its compounded impacts and WASH on self-reported physical and mental health of adults in the Vietnamese Mekong Delta.

Members of the local community involved in this research are

**Non-human subjects research using specimens/ animals collected as part of the study, or those housed in archival collections. Examples include archaeology, paleontology, botany and zoology.**

Did the permission you obtained from a local authority to perform the study include an agreement on access to outputs and benefit sharing? This may include procedures to enable fair distribution of the benefits and resources arising from the research performed. Please include any details of Prior Informed Consent and Benefit Sharing Agreements obtained. These may be required by field-specific regulations, for example the Convention on Biological Diversity (CBD) and the associated Nagoya Protocol.

The permission we obtained from local authority to perform the study did not include an agreement on access to outputs and benefit sharing.

If the material used in your study was imported, please A) provide the year it was imported and B) indicate whether permits were obtained to import/export the materials used, C) provide details of any permits obtained. If this information is not available, please indicate this.

N/A

If you used archival specimens, please state how the material used in your study was acquired by the institute it is held in and provide details of any permits obtained for the original excavations/ sample collection. If this information is not available, please indicate this.

N/A

How was the potential cultural significance of the materials collected in your study to local communities considered in your research design? Were Indigenous peoples and/or local researchers and institutions involved with archaeological excavations / collection of specimens? If so, please provide a description of their involvement.

N/A

If your manuscript includes photographs of human remains please indicate whether authors obtained permission from descendants or affiliated cultural communities to do so.

N/A
