# Supplementary material for: Household water and food insecurity negatively impacts self-reported physical and mental health in the Vietnamese Mekong Delta
Source: PLoS One. 2022 May 5;17(5):e0267344. doi: 10.1371/journal.pone.0267344 (PMC9071150; doi:10.1371/journal.pone.0267344)
Supplement: S1 Table — (DOCX) [file pone.0267344.s002.docx]

Supplemental Table 1. Details of drinking water source (DWS) by population group (n=552)

|  | n | % | NP Kinh % | SP Kinh % | Khmer % |
| --- | --- | --- | --- | --- | --- |
| Primary DWS | | | | | |
| Bottled | 181 | 32.8 | 34.2 | 30.9 | 32.1 |
| Rainwater | 144 | 26.1 | 3.6 | 53.6 | 40.4 |
| Piped water | 122 | 22.1 | 32.4 | 12.5 | 11.0 |
| Creek/lake/stream/pond water | 83 | 15.0 | 29.8 | 0.0 | 0.9 |
| Tube-well or drilled well water | 22 | 4.0 | 0.0 | 3.0 | 15.6 |
| DWS type (JMP) ^a^ | | | | | |
| Improved | 469 | 85.0 | 70.2 | 100.0 | 99.1 |
| Unimproved | 83 | 15.0 | 29.8 | 0.0 | 0.9 |
| Treated drinking water before consumption | | | | | |
| Yes | 363 | 65.8 | 68.4 | 60.1 | 67.9 |
| No | 189 | 34.2 | 31.6 | 39.9 | 32.1 |

^a^ Improved DWS as defined by JMP includes piped water, bottled water, tube-well or drilled well water, and rainwater; Unimproved: surface water (stream/lake/pond/creek/river).
