# Supplementary material for: Household water and food insecurity negatively impacts self-reported physical and mental health in the Vietnamese Mekong Delta
Source: PLoS One. 2022 May 5;17(5):e0267344. doi: 10.1371/journal.pone.0267344 (PMC9071150; doi:10.1371/journal.pone.0267344)
Supplement: Attachment — Submitted filename: Response to Reviewers.docx [file pone.0267344.s003.docx]

| **Reviewers’ comments** | **Response** |
| --- | --- |
| **Reviewer 1** |  |
| My main remark regards the way in which the definition of 'water security' as meant in its broadest sense (rows 315 - 319) is interpreted in a very narrow meaning to just water for domestic uses and sanitation - for which WHO/UN have made their own indicators, also for monitoring SDG 6.1. For example, water plays a vital, possibly more important role than just drinking water, or all domestic uses, in food security/nutrition and related physical and mental health, including through irrigation of rice and fisheries/salt intrusion in VMD. | We recognize that there needed to be a tighter definition of ‘water security’ and how this has been used in this study. To cover this, we have used the term ‘Potable Water Security’ and added an additional statement primarily in the Methods that we only considered water use for drinking and cooking and an overall estimation of the amount of water used per person per day for domestic uses within the house. Our study examined the influence and links between water and food consumption, not other uses. We did not consider water security for other uses outside the home such as crop irrigation. We trust that the additional words provide more clarity. |
| Second, can the authors clarify what 'purchased' water is in table 1? What are the water quality problems with that, if any? Is purchased water only for drinking? | 'purchased' water is in table 1 = bottled water for drinking  Purchased water (bottled water) in the VMD is pretty cheap and has poor quality because of inadequate management ^(1)^. Specifically, authorities that are responsible for managing quality of bottled water lack equipment, lab, and human resources to supervise and control companies which produce this water source.  This has been made clearer in the text, in particular in Table 1 and in the Discussion. |
| What about the much higher volumes needed for other domestic uses? | In the VMD, people purchase water mainly for drinking and cooking. They utilize rainwater water and other water sources for other domestic uses such as bathing and washing.  Again, our study focused on the influence and links between water and food consumption in the household, and not other uses. The only role of water used for other domestic purposes in this study was a determination on the total volume of water used per person per day as an indicator on the availability of domestic water supplies and thus, influence the amount of water available for consumption and cooking. Words explaining this have been added to the paper. |
| Minor remarks row 37: been = between? row 107: further specify whether urban/rural? row 299: food = food insecurity? | We have checked and fixed these small errors. |
| table 2: last column: the Khmer are compared with the Kinh of the northern province. Yet, the Khmer live mainly in the southern province. Hence, wouldn't comparison with SP be more relevant here - keeping more factors constant? | We agreed with this suggestion and re-analyzed data which has modified in Table 2. We have also changed the text where necessary to reflect the outcomes of thisreanalysis. |
| And a suggestion: for the further exploration of pathways through which water security for domestic uses and food security/ nutrition are linked, see, for example, IFPRI, e.g. Claudia Ringler. | Thank you for this suggestion, in the interests of space we have kept just one reference for this point in lines 382-383 |
| Lastly, some philosophical thoughts from a sociological/institutional perspective, not for this paper, but more general. The SDGs already emphasize the multiple linkages between the SDGs. There is quite some qualitative literature about linkages between water for drinking and other domestic uses, and health, e.g., infant morbidity (with contestations, though). System thinking is promoted. However, cross-sectoral collaboration is notoriously difficult, as also well recognized in the SDGs. The authors' narrow interpretation of the broad concept of 'water security' is another case in point: even within the water sector, the silos between WASH and other (irrigation, water resource management, pollution, climate etc.) are rigid. Yet, evidence shows that in rural areas, water supply systems designed for domestic uses (for healthy absorption of nutrients) are, in reality, often also used for productive uses (for the intake of nutrients). Certainly for rural people with multi-faceted agrarian livelihoods, it is obvious that they need water for both domestic and often a range of productive uses. Hence, the proposed combined water and food security programs are easier said than done. | We agree with the reviewer’s comments. |
| **Reviewer 2** |  |
| 1. The drinking water treatment variable has three categories: point of use, outside treated and untreated. Yet, the untreated category includes a variety of sources with significantly different safety profiles. Within the WHO-UNICEF JMP system for categorise water sources - boreholes/tubewells are an improved source as when properly designed and maintain protect water for most sources of contamination, and hence treatment is often no required. Whereas, surface water is significantly more likely to be contaminated and is very risky when not treated. Hence, I don't think that current categorisation tells you much about the safety of water being consumed and therefore in its current design that particular variable does not tell us much about water security (which is what you are ultimately interested in). This calls into question the results connected to that particular variable in my mind (for example results on L232-3 and others). There are various other more minor concerns I have with that variable such as putting treated piped supply in with bottle water, and also the implication that POT is the highest outcome when we no of the many flaws in POT in terms of behavioural adherence. | We have purposely moved away from the WHO-UNICEF JMP definitions to reflect the local conditions and resident behaviour in the VMD. Although JMP-WHO defines tube-wells or boreholes and bottled water as improved water supply, these water sources in the VMD are under-regulated and there are concerns over their quality ^(2)^. Unfortunately, in the VMD, boreholes or tube wells may not be an improved source as they are not properly designed and maintained to protect water from contamination ^(2)^.  In rural areas of the VMD, more than half of the households with access to a piped-water supply did not use this source of drinking water due to perceived poor quality/quantity, high connection fees, and preference for other water sources ^(3)^. Also, due to a lack of finance for operation, management, and maintenance, most of the available water supply systems are out-of-date and unreliable ^(4)^. Very few water stations have adopted any new water treatment technology. As a result, many households in the VMD refuse to use water supplied by these stations because water quality does not meet the required standards promulgated by the Ministry of Health ^(3)^.  In general, the quality of the three main water sources (surface water, groundwater, and rainwater) in the Mekong River Delta is increasingly threatened by a number of factors. The surface water and groundwater are largely contaminated by overuse of agricultural chemicals and fertilizers in farming and industrial activities, salinity intrusion and aluminum soils ^(5)^. The threat to the quality of these water sources is further worsened because there is a shortage of wastewater treatment plants in the region ^(6)^ , meaning wastewater from both domestic use and industrial parks is directly discharged into rivers and canals. Thus, surface water and groundwater are largely polluted.  Our study indicated that boiling water prior to consumption was common in the VMD, irrespective of the original source of the water. Therefore, we believe it is important to capture this behaviour in the final classification of drinking water source. We acknowledge the reviewer’s comment about behavioural adherence to correctly treatwater in this way, however, we see this as less of a problem than using a classification based on the original water source, which is then subsequently boiled. Supplemental Table 1 has been added, along with relevant results in the main text, providing detail about the original water source.  We have added words in the Discussion to cover these points raised by Reviewer 2. |
| 2. The discussion about the conceptualisation of water security is also limited and does not engage sufficiently with the extremely deep literature in this area. For example, the HWISE initiative which has mapped out these key components before in many papers, but even more broadly any literature on monitoring water supply within development programs (including grey literature) can already tell you about the various components you mention. | The HWISE was developed for universal use in 2019. At the time of this current study being conducted (2018), the HWISE has not yet been developed. We note that while the very recent reported work from the HWISE group is starting to report on links between water and food security, they have not yet extended their studies to South East Asia (including Vietnam). Thus, our study adds additional important information to complement the work done by HWISE. We have added some words in the introduction to note this.  Also, to these comments from Reviewer 2, our study did investigate key dimensions of potable water insecurity including accessibility, availability, quality, use and reliability. However, we only reported significant results in this paper due to word limitation and to keep the paper concise. |
| I don't really see how you have developed any sort of novel conceptualisation of water security. | We did not try to make novel conceptualization of water security. We created drinking water treatment variable because there is a concern over the quality of most of water supplies in the study area.  Although piped water is considered an improved drinking water source by the Vietnamese Government and the WHO-UNICEF JMP, a study found that piped water in the VMD had exceeded recommended upper limits for microbial indicator bacteria (E. coli), pH, turbidity, chlorine, ammonia, iron and mercury ^(3)^. Similarly, the quality of bottled water services (both public and private) are under-regulated due to a lack of human resources, laboratory equipment and budget {Chau, 2016 #908}. However, the WHO-UNICEF JMP (2017) defines bottled water as an improved drinking water source in order to estimate the coverage of WaSH for the global population ^(7)^. The definition of improved drinking water for this water source at the national and household levels, however, should be not the same as the WHO-UNICEF JMP because the safety of this water source heavily depends on specific context of each area or country.  Our finding is novel for its assessment of measures to assess water quality in relation to health given the lack of research on household water insecurity especially in Vietnam and other LMICS. |
| 3. Relatedly, the links between WASH and food insecurity are well known and articulated normally from a WASH-nutrition perspective. The links between WASH and nutrition were linked to improving child health with the need to protect against infection as well as ensure nourishment to avoid childhood stunting and to support healthy children. Hence, reflecting back on that trend and the evidence base that underpins it would be help enrich your framing of this project. | We agree with the reviewer that the links between WASH and nutrition in relation to improving child health are well examined. This is noted in the introduction. However, in our study, we are interested in health of food preparation providers in the household (typically female adults), and by inference, the whole household. We are also particularly interested in mental health. There is a significant gap in this area which this study addresses. |
| 4. My final comment is about the "so what" of the paper. I thought overall the study seemed rigorous but I was left wondering why assess these interlinkages when we know (or can at least theorise) that they exist. It is a bit like when studying multidimensional poverty - many things are interlinking and correlating but studying that often doesn't really tell us anything useful. Hence, what does the paper really tell us about these relationships?  What are the implications for governments and others who need to help respond to these problems? | Although the inter-relationships between WASH and food security are well identified, these interlinks are predominantly examined at the global and national levels. Despite that, the Vietnamese government still have implemented individual programs aiming at either improving water security or food security to protect and promote health for people. There is little information on the inter-relationships between food security (FS) and water security (WS) at the household level and it may be in part a reason for a lack of integrated programs for improving simultaneously FS and WS in Vietnam. Therefore, our study is significant for the Vietnamese government in providing evidence to persuade policy makers to adopt joint dual-action policies for interventions that simultaneously address water and food insecurity to generate larger improvements in health, particularly mental health.  In addition the results show that there is a need to examine household food and water security at scales lower than national levels to ensure that at-risk populations also benefit from national improvements.  We have added to the text in the discussions to highlight the above. |
| Does it provide more precision or accuracy in how they relate? | Yes, it does. The finding clearly showed which dimension of water security being inter-related with household food security and how strong they are linked. The finding also showed their compounding effect on health. |
| Are there particular directions of causality that can identified? | Yes, the results show that there is a strong potential that potable water insecurity may be a determinant of health and can be linked with food insecurity. |

1.	Li L, Li CS & Wichelns D (2016) Assessing household willingness to pay for bottled water in rural areas of the Mekong Delta, Vietnam. *Water Resources and Rural Development* **7**, 36-49.

2.	Ministry of Natural Resources and Environment & Ministry of Agriculture and Rural Development (2013) Mekong Delta plan: Long-term vision and strategy for a safe, prosperous and sustainable delta (Kế hoạch Đồng bằng sông Cửu Long). <https://www.mekongdeltaplan.com/> (accessed 26 April 2017).

3.	Wilbers G-J, Sebesvari Z & Renaud FG (2014) Piped-water supplies in rural areas of the Mekong Delta, Vietnam: Water quality and household perceptions. *Water* **6**, 2175-2194.

4.	Nguyen TLC, Phan D & Kyncl M (2018) Water supply status in rural areas of the Mekong Delta and development measures. *GeoScience Engineering* **64**, 35-41.

5.	Chau NDG, Sebesvari Z, Amelung W *et al.* (2015) Pesticide pollution of multiple drinking water sources in the Mekong Delta, Vietnam: evidence from two provinces. *Environmental Science and Pollution Research* **22**, 9042-9058.

6.	Chau MN (2016) Joint donors' support to national target program for rural water supply and sanitation phase 3. <https://www.google.com/search?q=Joint+donors%27+support+to+National+Target+Program+for+rural+water+supply+and+sanitation+Phase+3&oq=Joint+donors%27+support+to+National+Target+Program+for+rural+water+supply+and+sanitation+Phase+3&aqs=chrome..69i57.3887j0j7&sourceid=chrome&ie=UTF-8> (accessed 20 August 2019).

7.	WHO/UNICEF (2017) Progress on drinking water, sanitation and hygiene: 2017 update and SDG baselines. <https://www.unicef.org/publications/index_96611.html> (accessed 25 April 2017).
